# Advances in Energy Harvesting Technologies for Wearable Devices

**DOI:** 10.3390/mi15070884

**Published:** 2024-07-04

**Authors:** Minki Kang, Woon-Hong Yeo

**Affiliations:** 1 George W. Woodruff School of Mechanical Engineering, Georgia Institute of Technology, Atlanta, GA 30332, USA; minki.kang@me.gatech.edu; 2 Wearable Intelligent Systems and Healthcare Center (WISH Center), Institute for Matter and Systems, Georgia Institute of Technology, Atlanta, GA 30332, USA; 3 Wallace H. Coulter Department of Biomedical Engineering, Georgia Institute of Technology and Emory University School of Medicine, Atlanta, GA 30322, USA; 4 Parker H. Petit Institute for Bioengineering and Biosciences, Institute for Robotics and Intelligent Machines, Georgia Institute of Technology, Atlanta, GA 30332, USA

**Keywords:** energy harvesting, wearable devices, self-powering, soft electronics

## Abstract

The development of wearable electronics is revolutionizing human health monitoring, intelligent robotics, and informatics. Yet the reliance on traditional batteries limits their wearability, user comfort, and continuous use. Energy harvesting technologies offer a promising power solution by converting ambient energy from the human body or surrounding environment into electrical power. Despite their potential, current studies often focus on individual modules under specific conditions, which limits practical applicability in diverse real-world environments. Here, this review highlights the recent progress, potential, and technological challenges in energy harvesting technology and accompanying technologies to construct a practical powering module, including power management and energy storage devices for wearable device developments. Also, this paper offers perspectives on designing next-generation wearable soft electronics that enhance quality of life and foster broader adoption in various aspects of daily life.

## 1. Introduction

In recent years, the rapid proliferation of wearable electronics has reshaped the landscape of modern technology, with significant implications for healthcare, fitness, communication, and entertainment [1,2,3,4,5,6]. These devices, ranging from smartwatches and fitness trackers to advanced medical sensors and augmented reality headsets, are becoming integral to our daily lives, providing convenient and beneficial functionalities including motion sensing, hemodynamic activity monitoring, and interactions between electronics and the human body through visual, audio, or tactile elements, along with wireless transmission [7,8,9,10,11,12,13,14]. The development of flexible, stretchable materials and electronics expands the applications by providing seamless and skin-conformable interfaces between humans and machines, which enables high-quality sensing of physiological signals and improves the comfort of wearing [15,16,17,18,19,20,21,22,23]. However, the widespread adoption and commercial viability of wearable technology are contingent on overcoming critical challenges related to power supply [2,7,8]. Traditional batteries, despite continuous improvements, impose substantial limitations on wearability, comfort, and continuous operation. They are often bulky and rigid and require frequent recharging due to limited access to electricity, which can be inconvenient and hinder the seamless integration of wearable devices into everyday life.

The search for alternative power sources has led to significant interest in energy harvesting technologies, which promise sustainable and autonomous power generation [1,24,25,26,27]. These technologies convert ambient energy from the human body or the environment into electrical power, reducing or potentially eliminating reliance on traditional batteries. By harnessing ambient energy—such as mechanical movements, body heat, chemical energy from body fluids, and light—these technologies can provide a continuous and reliable power source. This not only enhances the user experience by reducing the need for frequent recharging but also supports the development of more flexible, lightweight, and comfortable wearable devices.

Despite their promise, wearable energy harvesting technologies face several limitations that must be addressed to achieve their full potential [28,29,30]. First, the energy output from many harvesting technologies may be insufficient to power high-demand wearable devices consistently, necessitating advancements in both energy efficiency and harvesting capacity [28,31]. Next, most current research focuses on the characterization of individual harvesting modules and their ability to power specific devices. These studies often rely on monolithic and volatile energy sources, assuming particular usage scenarios that may not reflect the diversity of real-world conditions [29]. This narrow focus can limit the practical applicability of energy harvesting solutions in dynamic and variable environments where wearables are typically used. Lastly, integrating these energy harvesting technologies into wearable devices presents technical challenges in energy management and storage [32,33,34]. Efficiently converting and storing harvested energy, ensuring compatibility with various electronic components, and maintaining device flexibility and comfort are complex issues that require multidisciplinary approaches.

This review introduces recent technological developments and innovations in energy harvesting for wearable electronics. Given the necessity of multidisciplinary studies for practical applications of energy harvesting technologies to wearable electronics, a comprehensive overview of multiple energy harvesting methods and their diverse applications offers beneficial information to further establish milestones and connections of scattered technologies. We cover recent progress in a wide range of energy harvesters classified based on fundamental physical phenomena, their output power, and wearable applications, focusing on the evaluation of their practicality by comparison with the power demand of wearable electronic elements.

## 2. Energy Harvesting Technology and Wearable Applications

### 2.1. Overview

**Figure 1:**
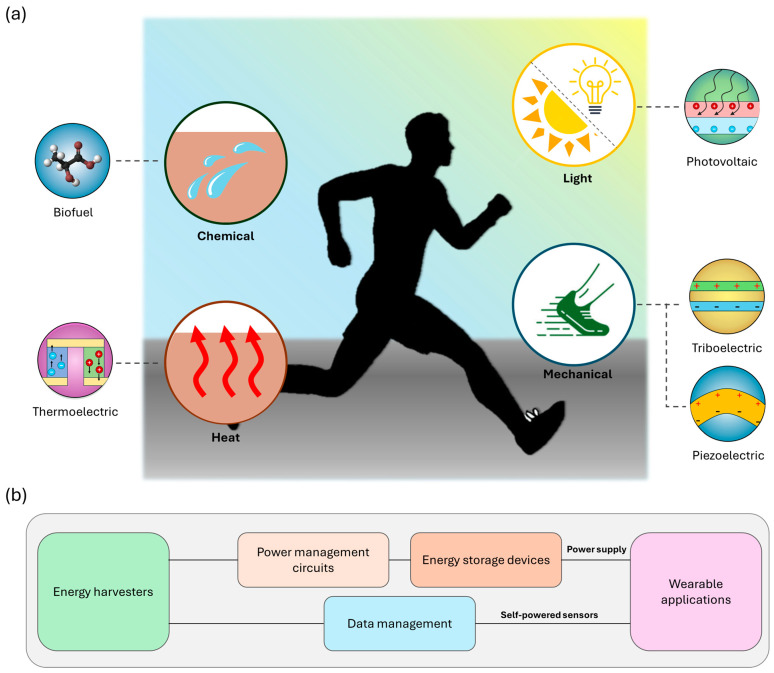
Concept illustration of wearable energy harvesting technology and applications. (**a**) Energy harvesting technologies classified by the energy source in the human body. (**b**) Block diagram of wearable applications of energy harvesters, including powering electronics and self-powered sensors.

Wearable energy harvesting technologies are increasingly critical for powering a range of portable electronic devices, leveraging diverse energy sources that exist in the human body (e.g., outdoor/indoor light, chemical energy of metabolic products, biomechanical energy, and body heat), particularly in the context of health monitoring and wearable sensors (Figure 1a). Among these technologies, photovoltaic (PV) cells are employed to harness energy from both indoor and outdoor light sources [35,36]. PV cells, capable of converting light into electrical energy, are advantageous due to their relatively high efficiency and the ubiquity of light. This makes them suitable for various applications, from smartwatches to fitness trackers, which can continuously recharge through exposure to light. Biofuel cells (BFCs) represent another innovative technology, utilizing chemical energy present in body fluids, such as sweat, to generate power [37,38,39]. These cells operate based on biochemical reactions involving enzymes or microbes, converting biochemical energy into electrical energy. BFCs are particularly promising for low-power devices integrated into clothing or directly onto the skin, as they offer a continuous and renewable energy source derived from the natural metabolic processes of the human body. Biomechanical energy harvesting, which includes piezoelectric and triboelectric technologies, captures energy from bodily movements [25,40,41]. Piezoelectric generators (PEGs) generate electricity as the displacement of ions within the crystal lattice creates an electric dipole moment when mechanical stress is applied to a piezoelectric material. Triboelectric generators (TENGs) produce power through the contact and separation of different materials. Both methods can effectively convert everyday movements into usable electrical energy, making them suitable for wearable devices that leverage the mechanical energy generated by activities such as walking, running, or even subtle gestures. Additionally, thermoelectric energy harvesting exploits body heat to produce electricity through the Seebeck effect, which converts temperature differences between the skin and the ambient environment into electrical voltage [42,43,44]. Not limited to the listed energy harvesting technologies, various promising energy harvesters such as moisture electric generators (MEGs) have been investigated. MEGs harness ambient moisture from the air to generate electricity through a process known as the hydroelectric effect [45,46,47]. This effect involves materials that absorb and release water vapor, creating a potential difference that can be converted into electrical energy. These generators show potential for applications in areas with high humidity, offering a renewable and sustainable energy source that complements existing technologies.

Wearable energy harvesting technologies have profound applications in powering electronic devices and self-powered sensors, particularly in the context of health monitoring, fitness tracking, and other portable electronics (Figure 1b). The output from these energy harvesters is typically converted and stored in energy storage devices, such as capacitors and batteries, using power management units (PMUs) [30,32,48]. PMUs are crucial for optimizing the harvested energy, ensuring efficient conversion, and managing the power distribution to wearable applications. For instance, photovoltaic cells integrated into smartwatches can continuously recharge batteries under ambient light, reducing the need for frequent manual recharging and extending the operational lifespan of the device. Table 1 summarizes various energy harvesting technologies’ working principles, energy source, optimal load, output power, and power supply [35,49]. When mechanical deformation is applied, TENGs and PEGs generate high-impedance instantaneous peak-to-peak AC power. Unlike other energy harvesters, which show almost immediate output generation, TENGs show a gradual ascending curve in their output when it starts to function after stopping for a while. This is because it takes a short time for surface charge to reach its maximum by repeated contact. PV cells and BFCs generate low-impedance DC power by imposing light and biofluid. However, the output varies by the intermittent source input and thus can be categorized as instantaneous. TEGs generate continuous, low-impedance DC output due to constant body heat, and the output power changes due to circumstance factors such as environmental temperature, activity, and physiological or pathological status. The lifetimes of energy harvesters depend on the types, structures, circumstances, and materials used. However, most show sufficient durability to maintain their output power for years.

Table 2 provides the power consumption of wearable electronics to provide guidance to assess the feasibility of powering applications of an energy harvester [27,50,51,52]. It is complex to ascertain whether energy harvesting technology can universally support wearable electronics due to the significant variation in power consumption based on their applications and designs. However, it appears feasible for low-power wearables with advances in output power and energy storage efficiency, yet further progress is required to power high-power wearables. For instance, as indicated in Table 2, OLEDs and LCDs demand hundreds to thousands of milliwatts, which far exceeds the output power range of energy harvesters shown in Table 1. Conversely, power consumption can be reduced from a few milliwatts to tens of microwatts when incorporating low-power sensors and microcontroller units (MCUs), which are sufficiently self-powered by wearable energy harvesters.

In addition to powering electronics, wearable energy harvesters can function as self-powered sensors [53,54]. These sensors do not require an external power supply, as they derive energy from the environment or the user’s body. For example, biofuel cells embedded in sportswear can generate electricity from sweat during physical activity and simultaneously perform the role of a sensor that monitors vital signs such as heart rate and hydration levels. Moreover, biomechanical and thermoelectric energy harvesters also find significant applications in self-powered sensors. Piezoelectric materials embedded in footwear can generate power from walking or running, enabling sensors to track steps, gait, and other physical activities [41]. Similarly, thermoelectric generators can be used in wearable health monitors to continuously measure body temperature and other physiological parameters by harnessing body heat [42]. These self-powered sensors are capable of providing useful signals through data management systems, facilitating continuous health monitoring, and promoting a more seamless integration of technology into daily life without the burden of frequent recharging.

### 2.2. Wearable Photovoltaic Energy Harvesting

**Figure 2:**
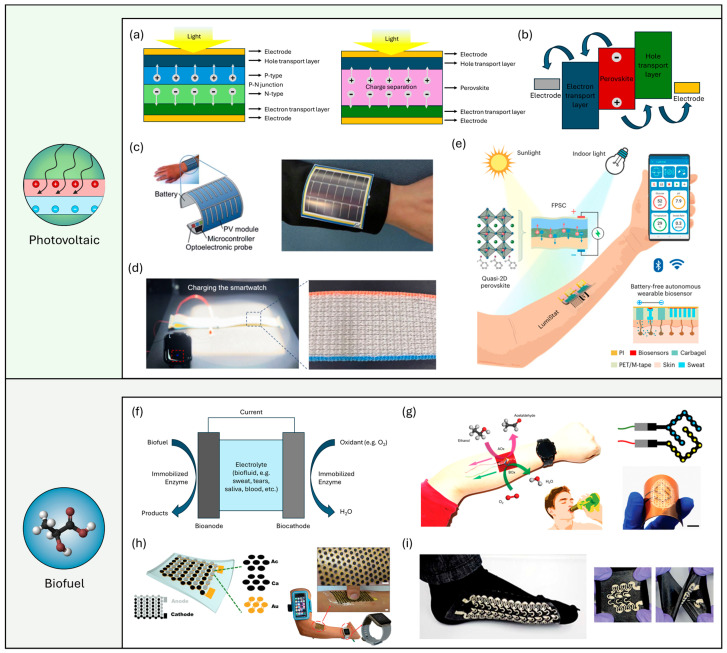
Wearable photovoltaic cells and biofuel cells. (**a**) Typical structure of PV cells and perovskite PV cells. (**b**) Energy band diagram of perovskite PV cells. (**c**) Illustration and photograph of a wristband containing a flexible silicon PV cell, a flexible battery, and a pulse oximeter component [59]. (**d**) Photographs of a wearable textile weaved with organic PV cells demonstrating powering a smartwatch [60]. (**e**) Schematic image of a wearable device that performs wireless multiplexed biomolecular analysis powered by a flexible PV cell [58]. (**f**) Schematic device structure and working mechanisms of BFCs. (**g**) Schematic of a BFC that harvests the chemical energy of ethanol in sweat [63]. (**h**) Schematic design and photographs of an electronic-skin-based biofuel cell based on a soft, stretchable substrate with its potential for wearable applications [37]. (**i**) Photographs of textile-based printable biofuel cells [64].

Wearable photovoltaic (PV) energy harvesting represents a rapidly growing field in sustainable technology, integrating the principles of photovoltaic conversion with the convenience of wearable devices. Significant advancements have been made in photovoltaic technology, leading to the development of flexible and lightweight materials suitable for wearable applications [55,56,57]. The fundamental physics of photovoltaic cells involves the conversion of sunlight directly into electricity via the photovoltaic effect (Figure 2a). Conventional silicon PV cells consist of a layered structure: a top contact grid and antireflection coating, a passivation layer, an emitter (n-type) doped with phosphorus, a base (p-type) doped with boron, a back surface field (BSF) to minimize recombination, and a back contact for electron collection. When photons strike the cell, they excite electrons in the semiconductor material, creating electron–hole pairs. The built-in electric field at the p-n junction separates these charges, generating a current when connected to an external circuit. In contrast, perovskite PV cells feature a perovskite absorber layer sandwiched between hole transport and electron transport layers, and typically methylammonium lead iodide. Excitons formed in the perovskite layer quickly dissociate into free electrons and holes due to efficient charge carrier properties. Electrons move through the electron transport layer towards the cathode, while holes migrate through the hole transport layer towards the anode, thus generating an electric current. The energy band diagram of perovskite PV cells illustrates the alignment of energy levels across these layers, which is crucial for facilitating charge separation and efficient electricity generation. Compared to other energy harvesting technologies, such as thermoelectric or piezoelectric systems, wearable PV devices offer significant advantages [55,56,58]. These include higher energy conversion efficiency, especially under direct sunlight, and the potential for continuous energy supply during daylight hours. Additionally, the integration of flexible and lightweight photovoltaic materials into textiles and wearable electronics enhances user comfort and device wearability. In the early stage of development, flexible silicon solar cells were introduced. Benefiting from the high natural abundance, excellent reliability, and high power conversion efficiency (PCE), such solar cells have long dominated the wearable photovoltaic market. In contrast to early rigid SSCs, flexible ones have been processed in recent years by depositing silicon on flexible substrates or embedding microscale SSCs into flexible substrates. Ostfeld et al. designed a flexible power source by integrating commercial SSCs with lithium-ion batteries (Figure 2c) [59]. Under an irradiance of 100 mW·cm^−2^, the self-charging system can be charged to 4.1 V at a current of around 40 mAh and demonstrates good stability during the cycles of charge and discharge. Next, based on the rapid development of flexible substrates, electrodes, and photoactive layers for PV cells, various PV cells, including organic solar cells, dye-sensitized solar cells, and perovskite solar cells, have been investigated as promising candidates for wearable power solutions in the form of a film, textile, etc. [57]. Noticeably, textile or fibrous PV cells have attracted significant attention due to their excellent flexibility and wearability despite limitations in their PCE originating from the intrinsic low dielectric constant of organic materials. Lv et al. [60] reported a fiber-shaped organic solar cell that exhibits a PCE of up to 9.4% under AM 1.5 G irradiation conditions (Figure 2d). They utilized non-fullerene-acceptor (NFA)-based organic semiconductors as the light-harvesting materials and carbon nanotube (CNT) yarn or silver wire as the counter electrode to twine around the primary electrode. To demonstrate its potential for wearable applications, a wearable textile weaved with PV cells was fabricated to power a smartwatch. Lee et al. reported an organic PV cell in a flexible wire format [61]. The organic PV cell comprises two stainless steel wires in a transparent cladding. One is the primary electrode, and another is coated with a silver film that serves as a secondary electrode. They contact each other through the organic electron transport layer, photoactive layer, hole transport layer, and conductive cladding from the primary to secondary electrode. The PCE of the wires ranges from 2.79% to 3.27%, which is relatively low compared to conventional Si-based PV cells. In another instance, Li et al. reported a carbon nanotube (CNT) fiber-supported double-twisted perovskite PV cell that exhibits a maximum PCE of 3.03% [62]. Next, Min et al. investigated a wearable sweat sensor powered by a quasi-two-dimensional perovskite solar cell module based on a flexible substrate that exhibited extremely high PCE exceeding 31% in indoor light circumstances (Figure 2e) [58]. The PV cell utilizes a p-i-n architecture and comprises flexible polyethylene terephthalate (PET) coated with indium tin oxide (ITO). Such high PCE is achieved by utilizing a quasi-2D PV cell, which has a narrower emission spectrum that matches the common indoor lighting sources such as LED, resulting in reduced sub-bandgap relaxation and recombination losses. The researchers demonstrated feasible wearable applications for multimodal physicochemical data acquisition (glucose, pH, sodium ion, sweat rate, and skin temperature) by integrating the PV cell with a biosensor array, microfluidic layer, and flexible electronic circuits.

### 2.3. Wearable Biofuel Cell

A wearable biofuel cell (BFC) is a device that generates electrical energy through biochemical reactions, typically involving enzymes or microorganisms that catalyze the oxidation of biofuels, like glucose or lactate present in body fluids (e.g., perspiration, blood) [38,65]. Fundamentally, it operates on the principle of converting chemical energy directly into electrical energy, similar to traditional fuel cells but using bio-compatible materials and processes. These devices typically consist of bioanodes and biocathodes separated by an electrolyte reservoir containing the biofluid as an electrolyte (Figure 2f). At the bioanodes, immobilized enzymes catalyze the oxidation of biofuels (e.g., glucose, lactate) present in the biofluid, generating electrons and protons. The released electrons travel through an external circuit, producing electrical power, while protons migrate to the biocathode. Simultaneously, at the biocathode, a different immobilized enzyme catalyzes the reduction of an oxidant (e.g., oxygen) from the air or dissolved in the biofluid, combining electrons and protons to form water as a byproduct. This redox process completes the circuit, facilitating continuous energy conversion. These cells can be classified as enzymatic biofuel cells, which use enzymes as catalysts, and microbial biofuel cells, which use living microorganisms [27,39]. Wearable biofuel cells offer several advantages, including their ability to harvest energy from naturally occurring bodily substances, providing a continuous and renewable power source for low-power wearable devices. They also produce minimal waste and are environmentally friendly. However, they face challenges such as lower power density compared to other energy harvesting technologies like solar or thermoelectric generators, potential issues with biofouling, and the stability and lifespan of the biological catalysts. Despite these drawbacks, the integration of biofuel cells into wearable technology presents a promising avenue for self-sustained, eco-friendly energy solutions. For the wearable application of biofuel cells for powering electronics, elevating the output power is crucial. Yu et al. investigated an extremely high-output flexible perspiration-powered electronic skin that harvests energy from human sweat through lactate biofuel cells for in situ monitoring of metabolic biomarkers [65]. The device consists of a flexible electrochemical patch comprising a BFC array and a biosensor array and a flexible electronic patch comprising rigid electronics on an ultrathin polyimide substrate. Lactate is one of the most promising metabolic products for energy harvesting for powering skin-interfaced electronic devices since it is present in sweat in tens of millimoles. The researchers addressed the limited power density and short lifetime by fabricating a BFC that consists of lactate oxidase immobilized bioanodes and Pt alloy nanoparticle decorated cathodes. As a result, the BFC array showed exceptionally high output performance with maximum open-circuit potential and output power of 0.6 V and 3.5 mW·cm^−2^ in 40 mM lactate solution, which is considered a record-high performance. Such effective energy harvesting allowed the powering of a biosensor array and Bluetooth module capable of monitoring glucose, urea, NH_4_^+^, and pH, and wireless communication, through management of harvested power by integrating a DC-DC boost converter and a capacitor.

Sun et al. [63] investigated a flexible BFC that harvests the chemical energy of ethanol/oxygen in sweat by leveraging epidermal microfluidic sweat sampling and a biocatalytic redox reaction (Figure 2g). Built on a flexible polyimide (PI) substrate and polyethylene terephthalate (PET) films, the BFC incorporates a skin-interfaced microfluidic module for on-body sweat transport, sampling, transfer, storage, and excretion. Also, multiple layers of three-dimensional coralloid nitrogen-doped hierarchical-micro-mesoporous carbon aerogels (3D-NHCAs), alcohol oxidase, and terephthalaldehyde (TPA) on a screen-printed array (SPA) were fabricated to prepare the bioanode, and multiple layers of 3D-NHCAs, bilirubin oxidase, and TPA were coated on other SPAs to form biocathodes. As a result, the peak output power reached 1.01 µW·cm^−2^ on the forearm. Next, researchers have made significant efforts to achieve device stretchability for seamless integration into the human body by utilizing flexible substrates and stretchable electrode interconnectors. Bandodkar et al. [37] proposed the use of a soft, flexible substrate combined with a hexagonal closely packed island–bridge architecture to mitigate the reduction in output power caused by gradual delamination due to mechanical mismatch, thereby achieving a high fill factor (~60%) that enhances output power (Figure 2h). This soft and stretchable BFC array exhibited a high output power of 1.2 mW·cm^−2^, demonstrating its potential for wearable applications through seamless skin integration. Furthermore, Jeerapan et al. [64] developed a highly stretchable and printable textile-based BFC (Figure 2i). The researchers employed stretchable carbon nanotubes (CNTs) and silver inks, along with a serpentine electrode design, enabling the BFC to maintain high stretchability and stable performance under strains as large as 100% for over 100 cycles.

### 2.4. Wearable Triboelectric Energy Harvesting

**Figure 3:**
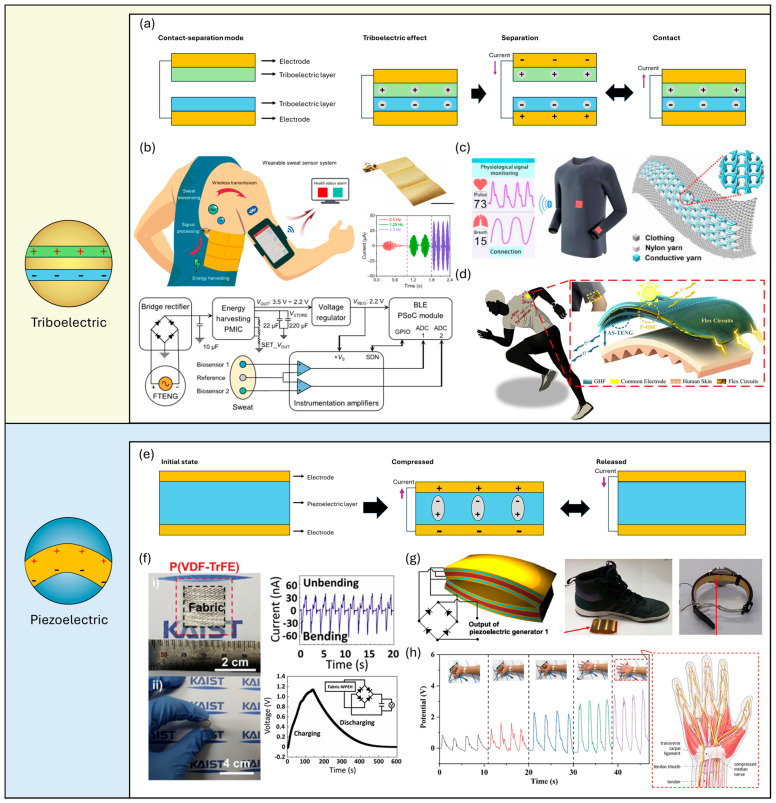
Wearable biomechanical energy harvesting. (**a**) Schematic structure and working mechanisms of contact–separation-mode TENGs. (**b**) Schematic illustration, photograph, output current, and block diagram of a freestanding-mode TENG-powered wearable sweat sensor [33]. (**c**) Schematics of physiological signal monitoring and structure of a triboelectric all-textile sensor array [67]. (**d**) Schematic illustration of the flexible hybrid energy harvester that consists of a TENG, an organic PV cell, and flexible electronic circuits [68]. (**e**) Schematic structure and working mechanisms of PENGs. (**f**) Photograph, output current, and 1 µF capacitor charging curve of a fabric-based wearable PENG [69]. (i) and (ii) demonstrate its dimensions and bendability, respectively. (**g**) Schematic structure and shoe insole and watch strap applications of curved multilayer PENG [70]. (**h**) Self-powered sensor based on nanowire PENGs to monitor finger movement [71].

Triboelectric nanogenerators (TENGs) are one of the most promising wearable energy harvesters due to their simple structure and high-output power. TENGs harvest biomechanical energy, including sliding, bending, stretching, and tapping motions, employing a variety of materials to suit a specific application [9,28,66]. TENGs generate a high peak-to-peak output power based on the triboelectric effect, where charge transfer occurs between any two different materials by contact and separation, coupled with electrostatic induction (Figure 3a). To be specific, when two different materials come into contact and then separate, they exchange electrons due to differences in their electron affinities, creating a charge imbalance. This contact and separation cycle can occur through various mechanical actions such as tapping, sliding, or rotating. The generated triboelectric charges induce an electrostatic potential difference when the materials move relative to each other, causing electrons to flow between electrodes connected to the materials. This electron flow constitutes an electric current, which can be harnessed to power electronic devices or stored for later use. Besides the contact–separation mode, TENGs can operate in several other modes, such as sliding mode, where two triboelectric materials slide against each other, generating electricity through continuous friction; single-electrode mode, where a single triboelectric layer interacts with an electrode to produce a charge; and freestanding mode, where a triboelectric material moves freely relative to a fixed electrode, inducing charge transfer. The efficiency and output of TENGs can be optimized by selecting appropriate material pairs and designing the device structure to enhance contact and charge transfer. Given their inherent flexibility and extensive design freedom in material selection and form factors, TENGs can be positioned on various parts of the human body to capture biomechanical energy and power wearable devices. Song et al. [33] introduced a wireless, battery-free wearable sweat sensor powered by a freestanding-mode TENG placed on the side of the torso (Figure 3b). This TENG features a stator with interdigitated copper electrodes on a polyimide (PI) substrate coated with PTFE and a copper slider on a PI substrate, utilizing flexible printed circuit board (FPCB) technology. Unidirectional sliding induced by arm movement generates charge flow between electrodes, producing short-circuit currents (I_SC_) of 8.39, 19.11, and 42.25 µA at 0.5, 1.25, and 3.3 Hz, respectively, with a peak output power of 0.94 mW. The wearable sweat sensor system (FWS3) includes three-panel TENGs, a power management integrated circuit (PMIC), a low-dropout voltage regulator, two low-power instrumentation amplifiers, and a Bluetooth-programmed system-on-a-chip (BLE PSoC) module. The rectified output current from the TENG charges a capacitor managed by the PMIC, discharging at a voltage threshold to power the PSoC module and enable Bluetooth data transmission. During treadmill running at 9 km/h, the capacitor’s charging/discharging cycle ranged from 2.1 to 3.7 min, demonstrating the feasibility of triboelectric energy harvesting for continuous wearable sensor operation.

Next, textile-based TENGs demonstrate the high degree of design freedom inherent in TENG technology, allowing for innovative integration into fabrics [56]. This is enabled by the incorporation of conductive and triboelectric materials into various textile structures, such as fibers, threads, or woven fabrics. As a result, TENGs can be customized in terms of size, shape, and configuration to fit different wearable applications seamlessly. This adaptability ensures that textile TENGs can harness energy from a wide range of human motion, providing a versatile and unobtrusive power source for wearable electronics, sensors, and health monitoring devices. Chen et al. [72] reported a self-powered wearable triboelectric sensor designed for real-time monitoring of pulse and respiratory signals, utilizing textile TENGs knitted with conductive and nylon yarns in a full cardigan stitch (Figure 3c). This configuration interlocks the yarns in loop units, generating output voltage in response to external mechanical forces due to charge transfer during contact and separation of the conductive and nylon yarns. The sensor exhibited high-pressure sensitivity, with 7.84 mV·Pa^−1^ at low pressures (<4 kPa) and 0.31 mW·Pa^−1^ at high pressures (>4 kPa), and a rapid response time of approximately 20 ms under 1 kPa. The device demonstrated excellent repeatability and durability, maintaining performance over 100,000 pressure cycles and 40 washing tests. Integrated into a flexible cardigan stitch, it effectively sensed physiological signals such as respiration and arterial pulse, indicating its potential for applications in sleep monitoring. Hybrid energy harvesters enhance output power and enable multimodal energy harvesting for a stable power supply despite irregular inputs. Ren et al. [68] developed a hybrid device combining flexible organic solar cells (F-OSCs) and a single-electrode triboelectric nanogenerator (TENG) (Figure 3d). Integrated through a flexible power management circuit with a common electrode, the solar cells capture energy from the top, while the TENG harvests mechanical energy from contact with human skin. The device features a groove-shaped, transparent micro/nanostructured haze thin film (GHF) that boosts both surface charge density and light-trapping efficiency. This GHF improved the solar cell’s short-circuit current density by over 16% and increased the TENG’s open-circuit voltage and short-circuit current by 120% and 105%, respectively. The hybrid system efficiently collects solar and mechanical energy without interference, charging a 10 μF capacitor from 0 to 0.7 V in 400 s, outperforming standalone F-OSC and TENG devices.

### 2.5. Wearable Piezoelectric Energy Harvesting

Piezoelectric nanogenerators (PENGs) constitute a promising avenue for harvesting biomechanical energy, along with the triboelectric nanogenerator (TENG) [25,34,73]. Rooted in the piezoelectric effect, a fundamental phenomenon observed in select materials such as quartz, zinc oxide, and specific ceramics and polymers, PENGs harness mechanical stress to generate electric charges (Figure 3e). This effect arises from the inherent asymmetry within the crystal lattice structure of these materials, facilitating the separation of positive and negative charges when subjected to deformation. This displacement of charge centers creates an electric potential difference, thereby producing an electrical output. PENGs exploit this principle by integrating piezoelectric materials into flexible substrates, where electrodes collect the generated charges. Notable output characteristics of PENGs include high voltage output, impressive power density, and a wide frequency response range, rendering them suitable for diverse applications. Their advantages encompass high energy conversion efficiency, flexibility, scalability, and environmental compatibility, positioning PENGs as promising solutions for powering wearable electronics, biomedical devices, and sustainable energy harvesting systems. In wearable applications, flexible piezoelectric polymers and block copolymers such as polyvinylidene fluoride (PVDF) and poly(vinylidene fluoride-tetrafluoroethylene) (P(VDF-TrFE)) are extensively utilized [70,74]. These piezoelectric polymers demonstrate a notable piezoresponse to external forces attributed to their elevated crystallinity and dipole arrangement ratio, achieved through heat treatment. Moreover, their superior flexibility renders them highly suitable for integration with wearable electronics, thus presenting favorable characteristics for such applications. Kim et al. [69] explored a fabric-based wearable PENG that has a sandwich-like heterostructure of a P(VDF-TrFE) (75/25) and two conductive fabrics via simple fabrication of tape casting and hot pressing (Figure 3f). Upon the dipoles being electrically poled at a high voltage field and elevated temperature, the fabric-based P(VDF-TrFE) film showed a high piezoelectric d_33_ coefficient of −32 pC·N^−1^, and the maximum output voltage and current of the PENG by finger pressing/elbow bending was 4.5 V and 69 nA, respectively, comparable to those of previously reported P(VDF-TrFE), BaTiO_3_-P(VDF-TrFE) composite-based PENGs. With a noticeable durability that sustained its performance for 15,000 bending cycles, the PENG showed the maximum peak power of 16.83 nW·cm^2^ at 60 MΩ load resistance and charged a 1 µF capacitor via finger bending under around 3 Hz. Next, Jung et al. [70] investigated a curved piezoelectric generator for wearable applications exhibiting significantly high output voltage and output current of 120 V and 0.7 mA, respectively (Figure 3g). Such high outputs were achieved by integrating piezoelectric PVDF multilayers in a curved structure. The instantaneous power density was estimated to be 3.9 mW·cm^−2^, which led to exceptional charging performance of the PENG; it charged a 47 µF capacitor to 5 V in 10 s. They demonstrated an application for battery charging by integrating power management circuits and the PENG, in which the 28 cm^2^ PENG is placed on the insole, one of the largest biomechanical energy generators in the human body. Also, the high output power of the PENG inserted in a watch strap and on the chest was demonstrated, indicating its potential for comprehensive wearable applications. Wassem et al. [71] investigated a GaN:Mg/ZnO nanowire-based flexible PENG (Figure 3h). The Fermi-level pinning was implemented by a reduction in the diameter of the nanowires, leading to a reduction in free carriers in nanowires and a noticeable improvement in output. The maximum output voltage and current were 52 V and 23 µA, respectively, and they were further increased, reaching 66 V and 40 µA, by depositing a 10 nm thick ZnO shell on GaN:Mg NWs. Based on the high output performance, the researchers demonstrated the sensing of finger movement by a nanowire PENG mounted on the finger. PENGs exhibit promising potential for integration with other energy harvesting technologies, offering the prospect of synergistic enhancement in energy conversion efficiency. Lee et al. [74] presented a study on skin-conformable piezoelectric–pyroelectric hybrid energy harvesters, featuring micropatterned piezoelectric poly(vinylidene fluoride-tetrafluoroethylene) (P(VDF-TrFE)) as a piezo-pyroelectric layer, and a micropatterned polydimethylsiloxane–carbon nanotube (PDMS-CNT) composite and graphene nanosheets as flexible electrodes. This hybrid device, characterized by its thin and flexible form factors, inherently exhibits high compatibility with human skin, rendering it well suited for wearable applications. Through the application of stretching–releasing and heating–cooling cycles and their combinations, the hybrid energy harvester produced distinct and combined outputs. The authors elucidated a coupling effect between the piezoelectric and pyroelectric phenomena, wherein an increase in the total polarization of the film through stretching led to an elevation in the pyroelectric current by thermal gradient, and vice versa.

### 2.6. Wearable Thermoelectric Energy Harvesting

**Figure 4:**
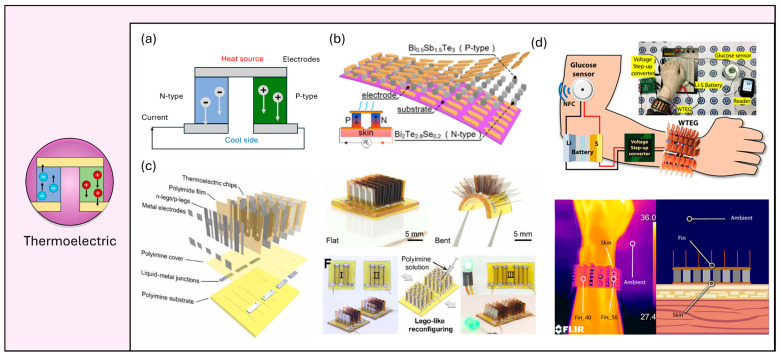
Wearable thermoelectric generators. (**a**) Schematic device structure and working mechanisms of TEGs. (**b**) Schematic structure of flexible TEG and self-powered wearable bracelet application [75]. (**c**) Schematic, photograph, and Lego-like reconfigurability of flexible, self-healable TEG [42]. (**d**) A self-powered glucose sensor powered by a wearable wristband TEG, a power management module, and a Li-S battery, and an IR image of the TEG demonstrating heat energy on the wrist [43].

Thermoelectric energy harvesters (TEHs) leverage the Seebeck effect to convert thermal energy into electrical energy, presenting a promising avenue for sustainable power generation [27,42]. The Seebeck effect is observed when a temperature gradient across a thermoelectric material induces a voltage difference, which can subsequently be harnessed to generate electricity (Figure 4a). This process involves connecting two dissimilar p- and n-type semiconductors to form a circuit with junctions maintained at different temperatures. The resultant temperature differential prompts charge carriers (electrons or holes) to diffuse from the hot junction to the cold junction, thereby generating an electric current. In the context of wearable applications, thermoelectric energy harvesters offer significant advantages due to their capacity to utilize the body’s intrinsic heat to power electronic devices. This capability facilitates the continuous operation of low-power electronics, such as health monitors and fitness trackers, without reliance on external batteries. Compared to other energy harvesting technologies, such as photovoltaic, chemical, or biomechanical energy harvesters, thermoelectric devices are less dependent on environmental conditions and movement, providing a more consistent and reliable energy source. Their compactness, durability, and ability to generate power from minimal temperature differences further underscore their suitability for integration into wearable technology, offering a convenient and environmentally friendly energy solution. Yuan et al. [75] proposed a high-output flexible thermoelectric energy harvester designed to power multisensory bracelets capable of continuously monitoring temperature, humidity, and human activity (Figure 4b). The energy harvester demonstrated a high output power of 3.5 µW·cm^−2^ and an output voltage range of 2.8–3.3 V without the need for an additional heat sink, even at body temperature in motionless and windless conditions. This excellent performance was achieved through a systematic optimization process using an object-oriented design method to match the internal resistance of the TEHs with the load resistance for power matching. This process involved manipulating the number of thermoelectric grains (n), the fill factor (f), and the series–parallel connection mode (m), based on computational simulations and experimental validation. To demonstrate its potential in wearable applications, the authors fabricated a wristband integrated with power management components, including an ultra-low voltage booster, a capacitor, and a low-power MCU. The sensory system utilizes a sleep–wake mode to optimize energy conservation and maintain sustainability in diverse conditions. During standby mode, the TEH’s power output is reduced to support only the accelerometer (5.4 μW) and LCD (9.5 μW), with any excess energy being stored in the capacitor. When in wake mode, the TEH provides power to the entire sensory system, which requires about 2 mW. One of the challenging drawbacks that limit the viability of TEHs in wearable applications is low-output voltage and consequent low energy storage efficiency, and there have been various attempts to increase the output voltage or match it with the potential window of a battery using power management circuits such as a buck boosting voltage regulator. Kim et al. [43] reported self-powered wearable wrist bracelet electronics for continuous glucose monitoring powered by a TEH (Figure 4c). In this study, the researchers aimed to minimize energy loss during voltage boosting, which arises from the low-voltage output of thermoelectric harvesters (TEHs) and the mismatch with the potential window of conventional batteries. To address this issue, they employed a Li-S battery, which has a charging voltage of approximately half that of Li-ion batteries. Ren et al. [42] introduced a TEH in which open-circuit voltage is extremely high, reaching 1 V·cm^−2^ at a temperature difference of 95 K (Figure 4d). Another highlight of this work is that the TEG system has high stretchability and Lego-like reconfigurability. These noticeable characteristics are realized by integrating high-performance modular thermoelectric chips on a thermoset polyimine substrate capable of self-healing by heat.

## 3. Power Management and Energy Storage

When it comes to wearable applications of energy harvesters, a power management unit (PMU) is essential to manage the power harvested, stored, and delivered to the devices. However, harvested energy is very variable depending on the environment and energy sources. PMUs for various energy harvesting technologies play a crucial role in efficiently converting and managing harvested energy for practical use. For DC forms such as PV cells, BFCs, and TEGs, PMUs typically include maximum power point tracking (MPPT) algorithms to optimize energy extraction from varying environmental conditions. PV cells convert sunlight into DC electricity, necessitating MPPT to match the varying solar intensity. BFCs and TEGs require voltage regulation circuits due to their low and fluctuating voltage outputs from biochemical reactions and environmental temperature changes. For AC forms such as TENGs and PENGs, PMUs might include MPPT and require rectifiers to convert AC to DC and capacitors for energy storage or voltage regulation. Typically, the outputs charge the capacitor, and the PMU detects the voltage of the capacitor and discharges it in DC to charge an energy storage device with a regulated voltage when the voltage of the capacitor reaches a predetermined threshold. In all cases, PMUs are designed to maximize efficiency, manage fluctuating input voltages, and store or deliver energy in a stable and usable form for electronic devices or grid integration. The low power level, dynamic power, and frequency of harvested energy increase the power consumption and complexity of a PMU [34,76]. For example, the output power of PV cells decreases in indoor conditions or at night and is largely changed by varying angle alignment with the light source. BFCs and TEGs also show high variability in their output depending on the metabolism of human subjects, the degree of physical activities, and the weather. The output generation of biomechanical energy harvesters is intermittent, solely depending on the occurrence of physical movement and physical parameters of human subjects, such as weight. Furthermore, the output power level of an individual energy harvester seems insufficient for powering wearable electronics. Therefore, the development of efficient power management for variable energies from multiple sources is crucial.

**Figure 5:**
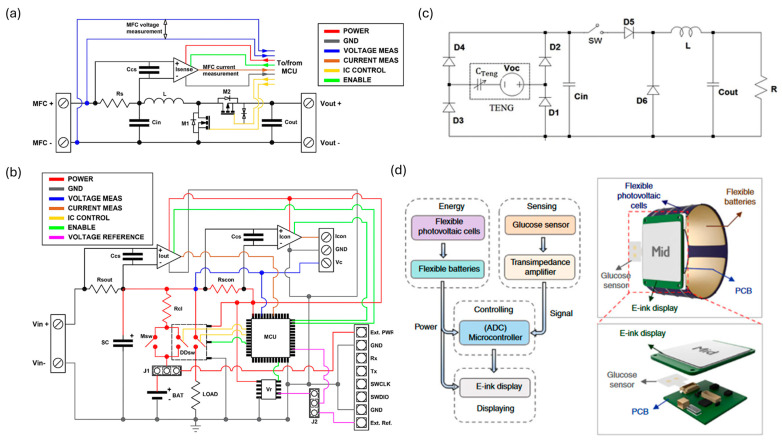
Power management modules for wearable energy harvesters. Circuit diagrams of (**a**) buck synchronous boost converter and (**b**) power management module for serially connected BFCs [77]. (**c**) Circuit diagram of power management module for TENGs. (**d**) Block diagram of PV cell-powered wireless glucose sensor [32].

As representative PMUs for DC outputs, Fernandez Feito et al. developed a power management system with a maximum power point tracking algorithm for multiple biofuel cells (Figure 4a,b) [77]. Biofuel cells exhibit low output voltages that reduce the energy storage efficiency, and serially connecting them can be an effective strategy to address the issue. However, this may result in voltage reversal due to extreme differences in output voltage among the connected cells, and this evokes the demand for the incorporation of maximum power point tracking (MPPT) control. The researchers employed a synchronous boost converter (Figure 5a). An ultra-low-power microcontroller and a current sense amplifier measure the voltage and current of the biofuel cells and amplify the voltage drop across the shunt resistor by 200 (Figure 5b). For voltage regulation, a digital dual switch was used, and it can connect the supercapacitor to a power supply to charge it to a predetermined voltage level. For MPPT control, a variable step size incremental conductance method was employed, and the N-channel MOSFET duty cycle was modulated to improve the system’s output power response. As a result, efficiencies of up to 87% were achieved. Next, as representative PMUs for AC outputs, Harmon et al. designed a power management system based on a buck converter for TENGs (Figure 5c) [78]. They utilized a silicon-controlled rectifier and Zener diode to control the power flow instead of using a MOSFET and logic ICs to reduce the power consumption of the power management module. TENGs have high capacitive internal impedance ranges around megaohms, and conventional power management is not suitable. To address this, they controlled the energy flow through four phases by leveraging switches—energy flows from the TENG to an in-capacitor, a set of inductors, a resistor, and an out-capacitor—and the load, respectively. As a result, the maximal efficiency of power transfer from the in-capacitor to the load and the overall energy conversion efficiency of the power management system reached 89.8% and 84.3%, respectively.

Alharwari et al. [76] designed a PMU for two energy harvesters, specifically a PENG and a TEG. The energy harvested by the PENG and TEG is stored in a storage capacitor via an energy combiner. For this purpose, the output of the PENG is rectified by a rectifier circuit to convert the AC signal to DC, and the voltage of the TEG output is boosted by a DC-DC converter. The PMU comprises two switched capacitor circuits that generate two regulated output voltages, which are 0.6 V and 1 V. Moreover, a power manager is designed to generate control signals that manage different modes of operation for the biomedical processor. The PMU employs dynamic voltage scaling (DVS), multiple voltage domains, and power and clock gating to minimize power consumption. For effective power management, they employed the active and sleep modes of processors, in which current consumption was 100 µA and 10 µA, respectively, and execution time and sleep time were manipulated via regulated output voltage levels. Mendez-Lira et al. [34] designed a PMU to manage harvested energy from a glove-type garment PENG that converts the mechanical energy from finger movement. The PENG is connected to a full-wave rectifier bridge, over-voltage protection, an energy storage capacitor (68 μF/25 V), and a PMU. During the former, the capacitor is isolated from the load while being charged using the energy harvested from the fingers. During the latter, the stored energy is transferred to the load, making the capacitor discharge gradually. Yu et al. [65] managed the harvested power from a BFC array by integrating a boost converter, instrumentation amplifiers, and a programmable system-on-chip (PSoC) module. The DC-DC boost converter amplifies the output voltage of the BFC array with minimal power loss (~20%), and the boosted output of 3.3 V charges a 660 µF capacitor. The Bluetooth module periodically wakes up from sleep mode to effectively maintain the stored energy level and enable data processing in an analog–digital converter (ADC) during sleep mode and wireless data transmission via the Bluetooth module in awake mode. Zhao et al. [32] introduced a self-powered and fully integrated smartwatch powered by PV cells (Figure 5d). Utilizing the high output voltage and DC power, the PV cells directly charge a flexible battery. The wristband of the assembled smartwatch functions as the power supply module, consisting of two flexible photovoltaic cells connected in parallel for efficient energy harvesting, with three flexible batteries connected in series on the opposite side, capable of being charged up to 6.0 V.

## 4. Challenges and Perspectives

### 4.1. Output Power

One of the primary challenges in wearable energy harvesting technologies is the optimization of output power [25,30,72]. Improving energy conversion efficiency is crucial, as it directly impacts the amount of usable energy generated from the source. Optimal device structure design is essential to maximize energy capture and minimize losses. Hybrid energy harvesting, which involves integrating multiple types of energy harvesters, can enhance overall efficiency by tapping into various energy sources simultaneously, such as combining photovoltaic cells with piezoelectric or thermoelectric generators [68,74,79]. Future research should focus on developing advanced materials and innovative designs that boost energy conversion rates and output power.

### 4.2. Miniaturization

The trend toward miniaturization in wearable technology poses significant challenges, particularly in maintaining mechanical integrity and consistent energy conversion efficiency [26]. Small-scale devices often suffer from high output fluctuation due to varying environmental conditions and the limited surface area for energy capture. Developing robust, flexible materials that can withstand mechanical stress without compromising performance is vital. Additionally, innovative design strategies are needed to stabilize energy output and improve efficiency in miniaturized devices.

### 4.3. Durability and Wearability

Ensuring durability and wearability in energy harvesting devices involves using flexible materials and structures that can endure repeated mechanical deformation while maintaining performance [73,75]. Minimizing performance degradation over time is crucial for the long-term viability of wearable devices. Standardizing the manufacturing process to produce reliable and high-quality energy harvesters can help address these challenges. Future advancements should aim at creating materials and designs that offer both flexibility and durability, ensuring that devices remain functional under various conditions.

### 4.4. Power Management

Effective power management is essential for optimizing the performance of wearable energy harvesters [28,76]. Managing power from multiple sources requires sophisticated algorithms and hardware to balance energy input and output efficiently. Minimizing power consumption through efficient power point detection and sleep–wake operation can significantly extend the operational lifespan of wearable devices. Innovations in power management units (PMUs) that can handle irregular energy input and optimize energy storage will be critical for future developments.

### 4.5. Self-Powered Sensors

Wearable self-powered sensors are pivotal for real-time health and environmental monitoring [53]. Ensuring accurate performance calibration and minimizing the effects of environmental variables are significant challenges. These sensors need to operate reliably without an external power supply, which requires advanced energy management and storage solutions. Future research should focus on enhancing sensor accuracy and stability, ensuring they provide consistent and reliable data in various conditions.

### 4.6. Multifunctional Integration

Integrating energy harvesters with other electronic components, such as batteries, circuits, sensors, and communication modules, presents both challenges and opportunities [27]. Optimizing the packaging structure to accommodate multiple functionalities while maintaining a compact and lightweight design is crucial. Achieving seamless integration without compromising the performance of individual components requires innovative engineering and design approaches. Future perspectives should aim at developing multifunctional systems that enhance the overall functionality and user experience of wearable devices.

## 5. Conclusions

Wearable energy harvesting technologies hold immense potential for revolutionizing the field of portable wearable electronics, self-powered sensors, and intelligent systems. By harnessing various energy sources such as light, body fluids, biomechanical movements, and body heat, these technologies can significantly reduce the dependency on conventional power supplies, enhancing the sustainability and convenience of wearable devices. Despite the numerous challenges, the ongoing work in power optimization, miniaturization, soft material engineering, nanomaterial printing, and power management strategies is paving the way for developing next-generation wearable soft electronics. Future research and development efforts should focus on overcoming these hurdles to fully realize the potential of energy harvesters, leading to more versatile and autonomous wearable devices seamlessly integrating into daily life.

## Figures and Tables

**Table 1:** Overview of wearable energy harvesting technologies [35,49].

Name	Working Principle	Energy Source	Optimal Load	Output Power (mW·cm^−2^)	Power Supply
Piezoelectric	Piezoelectric effect and electrostatic induction	Biomechanical energy	kΩ~MΩ	<10^−2^	Instantaneous AC
Triboelectric	Triboelectric effect and electrostatic induction	Biomechanical energy	MΩ	10^−2^~10^−1^	Instantaneous AC
Thermoelectric	Seebeck effect	Heat energy	Ω	<10^−3^	Continuous DC
Photovoltaic	Photovoltaic effect	Sunlight	Ω	1~10	Instantaneous DC
		Indoor light		10^−3^~10^−1^	
Biofuel	Biomolecule catalytic redox	Biochemical energy	Ω	10^−2^~1	Instantaneous DC

**Table 2:** List of elements in the power consumption of wearable electronics [27,50,51,52].

Name	Classification	Power Consumption
Data processing	MCUs/SoCs	0.507 µW~216 µW
	ADC/DAC	15 µW~1.95 mW
Low-power sensors	ECG	>5.2 mW
	PPG	68 µW~4.8 mW
	Temperature	82 µW~241 µW
	Accelerometer	0.35~0.9 mW
Wireless transmission	Bluetooth, WiFi, NFC/RFID	25 µW~200 mW
Display	OLED, LCD	10^2^~10^3^ mW
	Low-power display	10^−3^~10^−1^ mW

## Data Availability

Not applicable.
